# Supplementary material for: Photon deceleration in plasma wakes generates single-cycle relativistic tunable infrared pulses
Source: Nat Commun. 2020 Jun 3;11:2787. doi: 10.1038/s41467-020-16541-w (PMC7271200; doi:10.1038/s41467-020-16541-w)
Supplement: Supplementary file 1 — Supplementary Information [file 41467_2020_16541_MOESM1_ESM.pdf]

Supplementary Information for

Photon deceleration in plasma wakes generates single-cycle relativistic tunable infrared pulses

Zan Nie *et al.*

Supplementary Figures

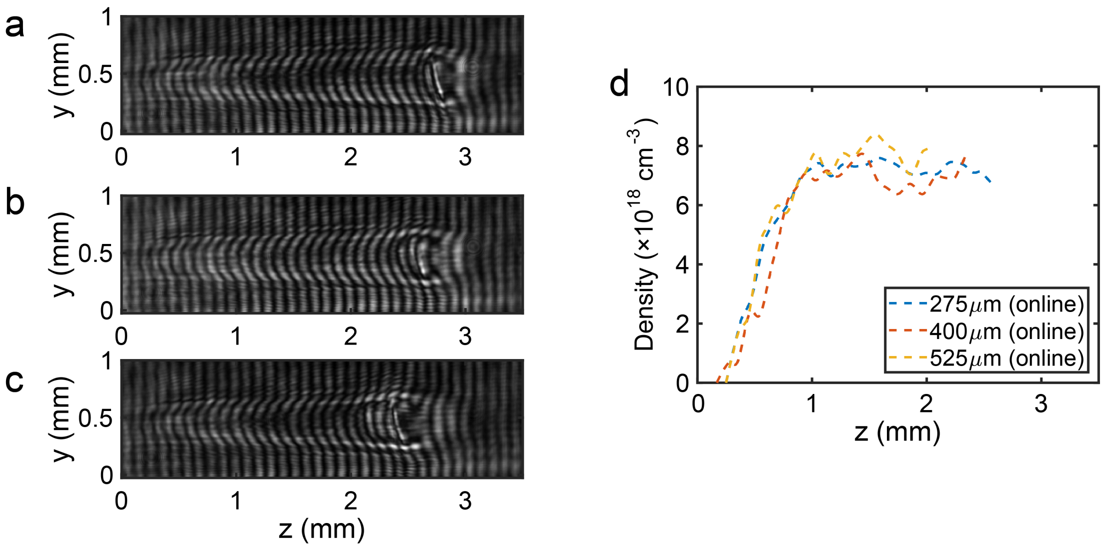

Supplementary Figure 1 Online density measurement. a-c, The online interferometry images in different cases (the blade inserted 275, 400, and 525 μm into the gas jet). **d**, The measured hydrogen plasma densities of the PC sections in different cases.

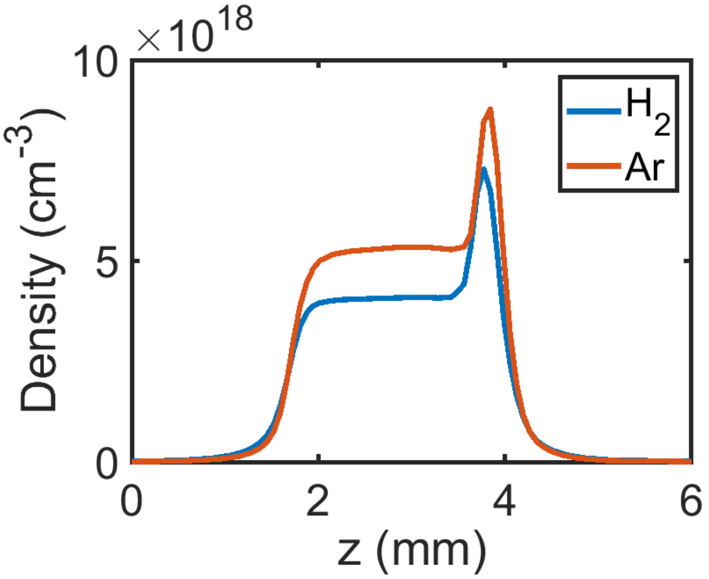

Supplementary Figure 2 Comparison of simulated gas density profiles of hydrogen and argon. In both cases, the blade inserts 400 μm into the gas jet with the same gas pressure.

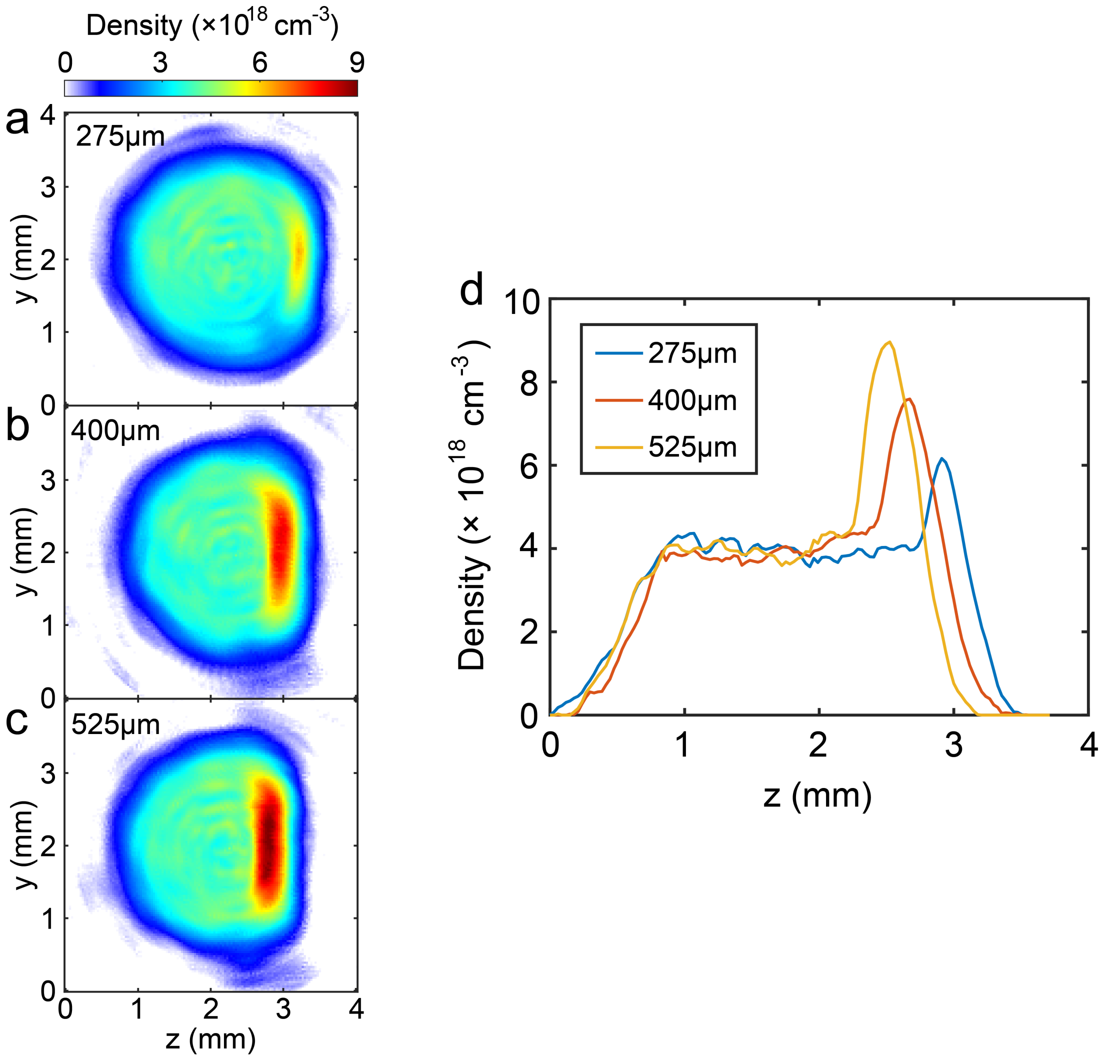

Supplementary Figure 3 Offline density measurement. a-c, The two-dimensional distributions of argon densities in different cases (The blade inserts 275, 400, and 525 μm into the gas jet). **d**, The on-axis density profiles of argon densities in different cases (The blade inserts 275, 400, and 525 μm into the gas jet).

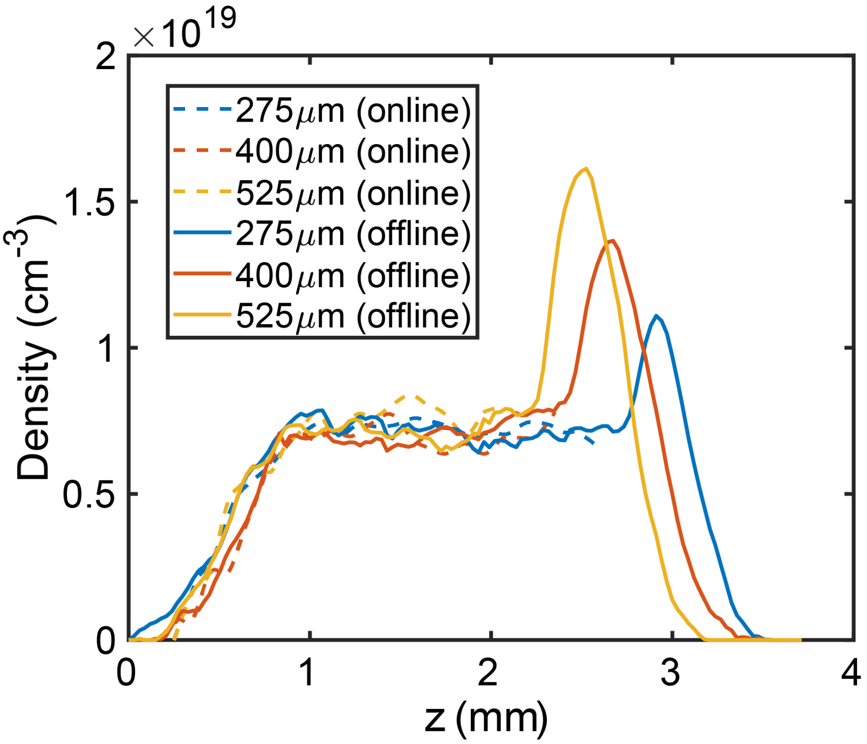

Supplementary Figure 4 Comparison of online and corrected offline densities in different cases (The blade inserts 275, 400, and 525 μm into the gas jet). The dashed lines represent online densities. The solid lines represent corrected offline densities.

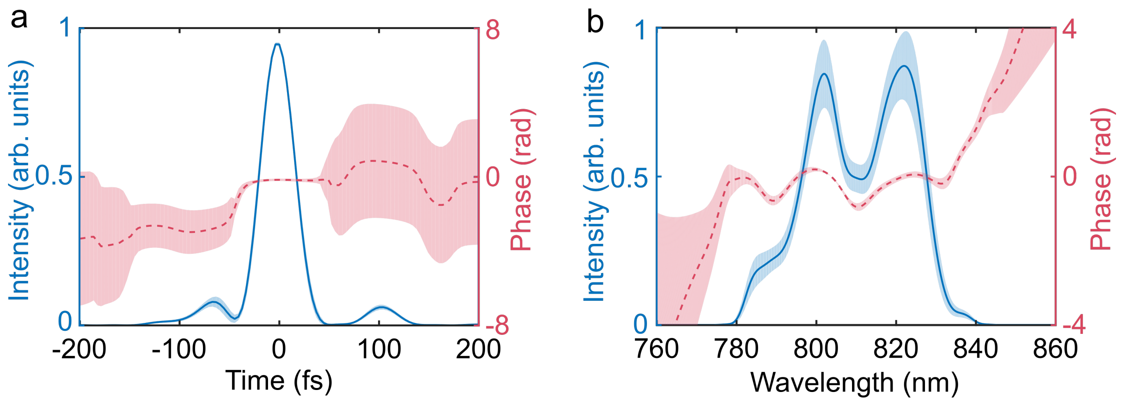

Supplementary Figure 5 The measured reference pulse (60 shots). a, The temporal intensity and phase. The measured reference pulse duration (FWHM) is around 40 fs. **b**, The spectral intensity and phase. The central wavelength of the reference pulse is about 810 nm. The shaded region represents the standard deviation.

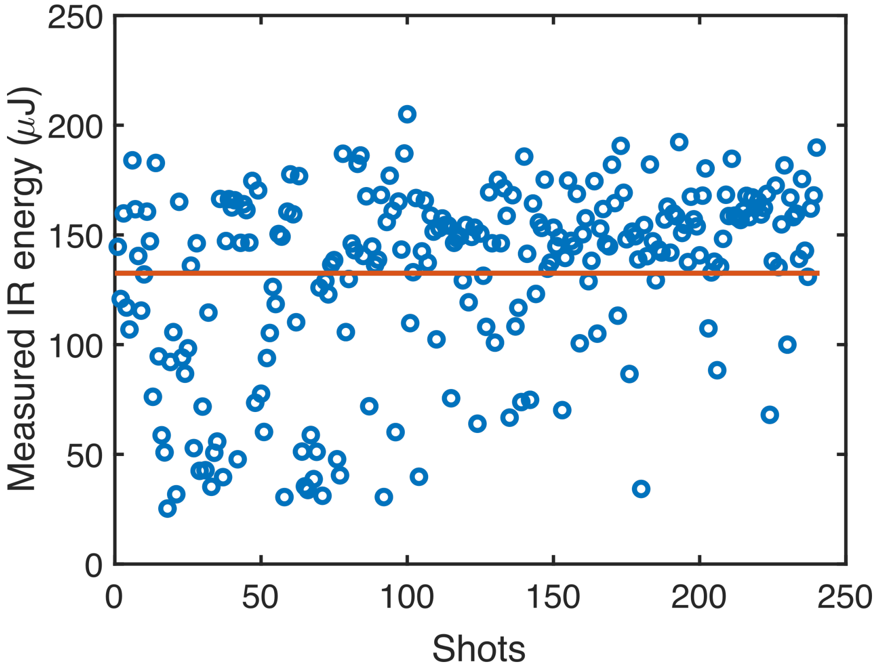

Supplementary Figure 6 Measured IR energy (240 shots). The orange line represents the average value.

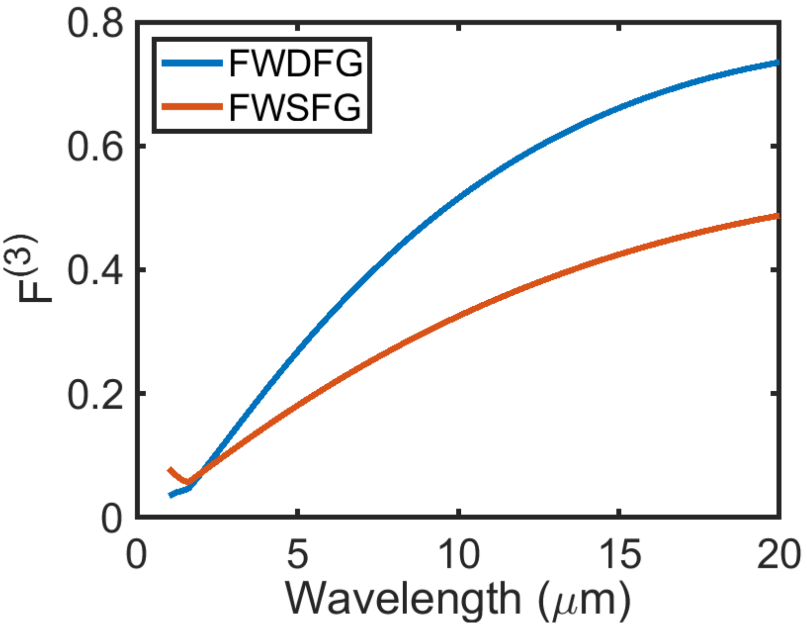

Supplementary Figure 7 Phase-matching functions $F^{(3)}$ for FWDFG and FWSFG processes. $F^{(3)}$ linearly scales with four-wave mixing conversion efficiency. In most wavelengths except below 2 μm, FWDFG process has larger conversion efficiency than FWSFG process.

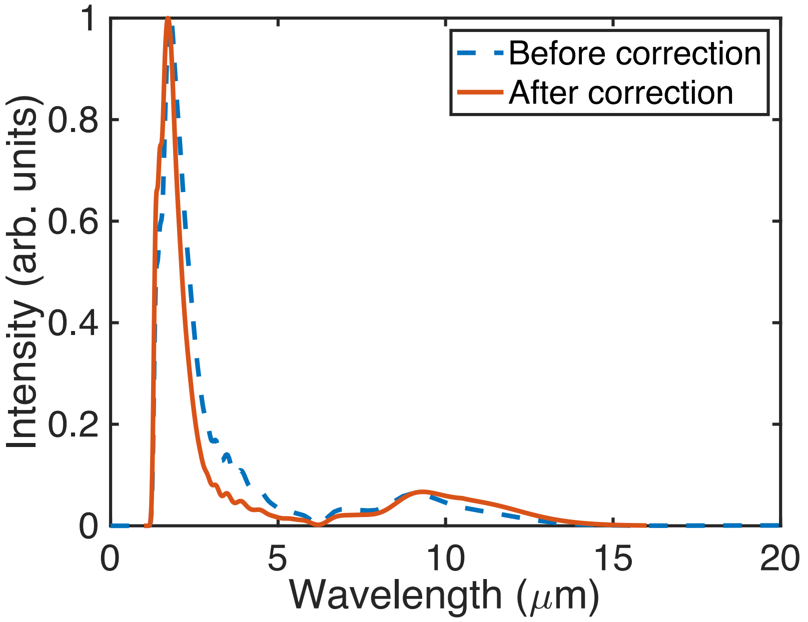

Supplementary Figure 8 Comparison of normalized IR spectra before and after correction. The measured spectra are filtered by 6 pieces of ITO-coated beam splitters in the experiment so that they do not have spectral component below 1.5 μm.

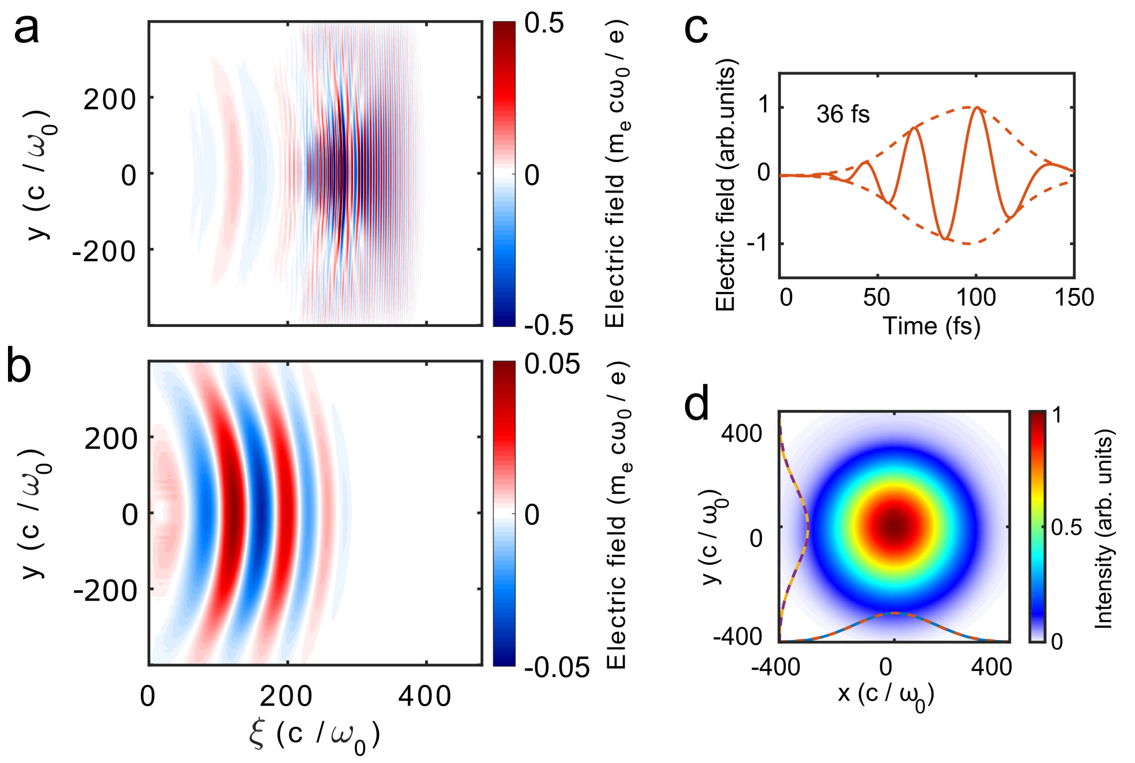

Supplementary Figure 9　The simulated temporal and spatial properties of the LWIR pulse for the case of the blade inserting 400 μm into the gas jet. a-b, The transverse electric field of the original and filtered LWIR (6-20 μm) pulse after exiting the plasma structure. **c**, The on-axis transverse electric field of the LWIR pulse. The pulse duration of the LWIR pulse (FWHM of intensity) is 36 fs. **d**, The beam profile of the LWIR pulse. The lineouts of the LWIR beam profile in both directions (solid) agree well with TEM_{00} mode (dashed). Here, $\xi = z - ct$, where z is propagation distance, and t is time. The x and y axis are transverse axes perpendicular to the drive laser propagating direction.

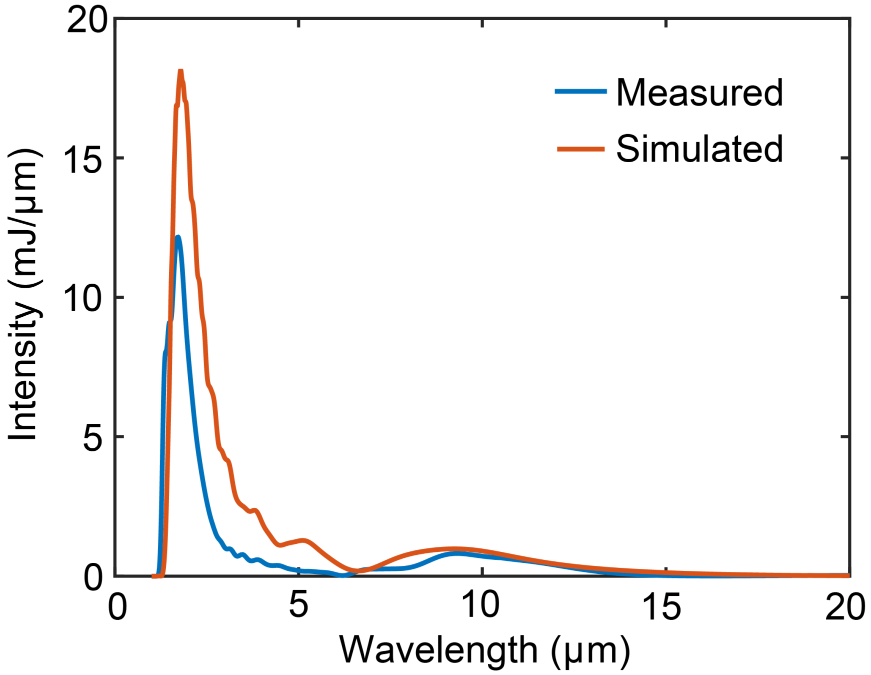

Supplementary Figure 10 Comparison of the measured and simulated spectra for the case of the blade inserting 400 μm into the gas jet. The simulated spectrum is filtered by 6 pieces of ITO-coated beam splitters just as in the experiment so that both spectra do not have spectral component below 1.5 μm.

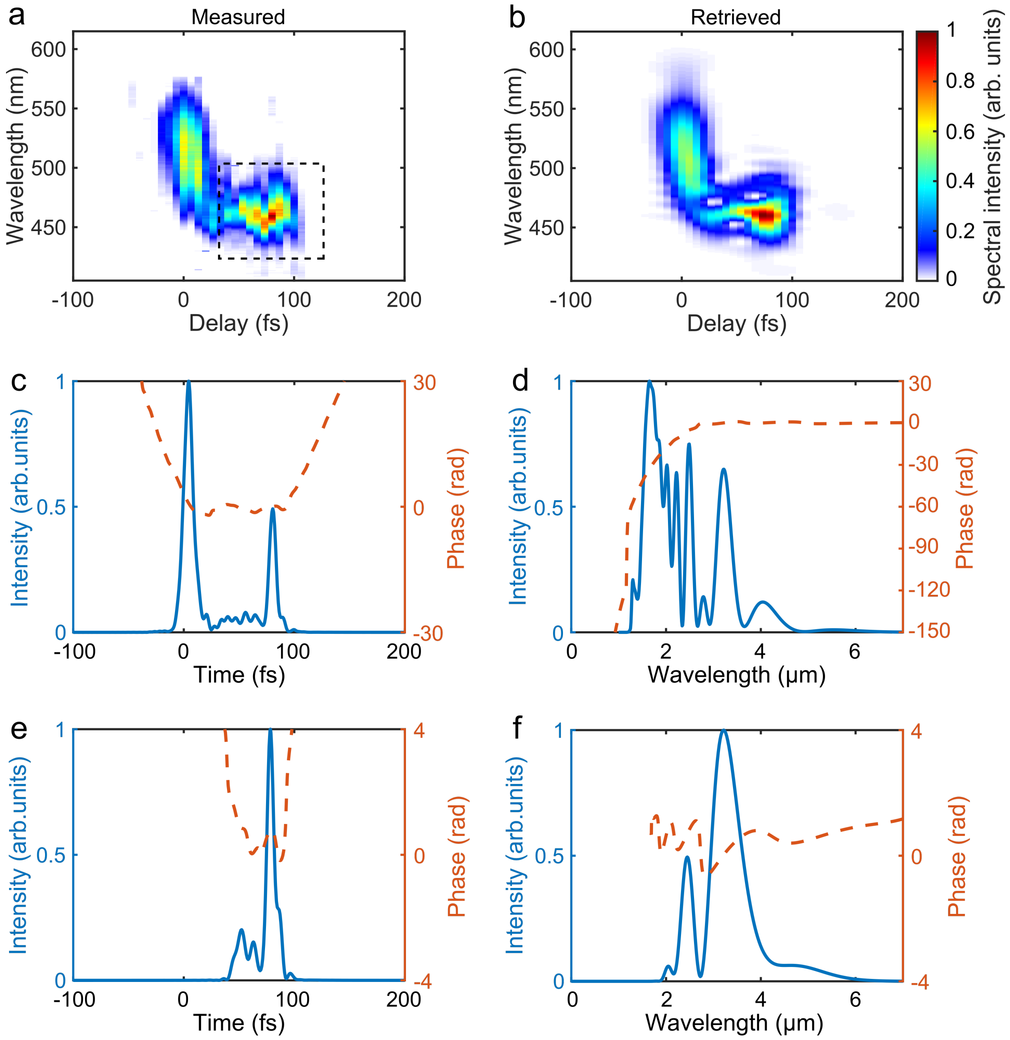

Supplementary Figure 11 The case with central wavelength of 3.2 μm. a,**b**, Measured and retrieved XFROG traces. **c**,**d**, Retrieved IR temporal/spectral intensity and phase. **e**,**f**, Retrieved temporal/spectral intensity and phase of the LWIR (dashed black box in (**a**)) pulse. The pulse duration (FWHM) of the LWIR pulse is 9.0 fs. The wavelength range (FWHM) of the LWIR pulse is 3.06-3.61 μm. The LWIR energy is 3.5 mJ. The estimated normalized vector potential $a_0 = 1.01$.

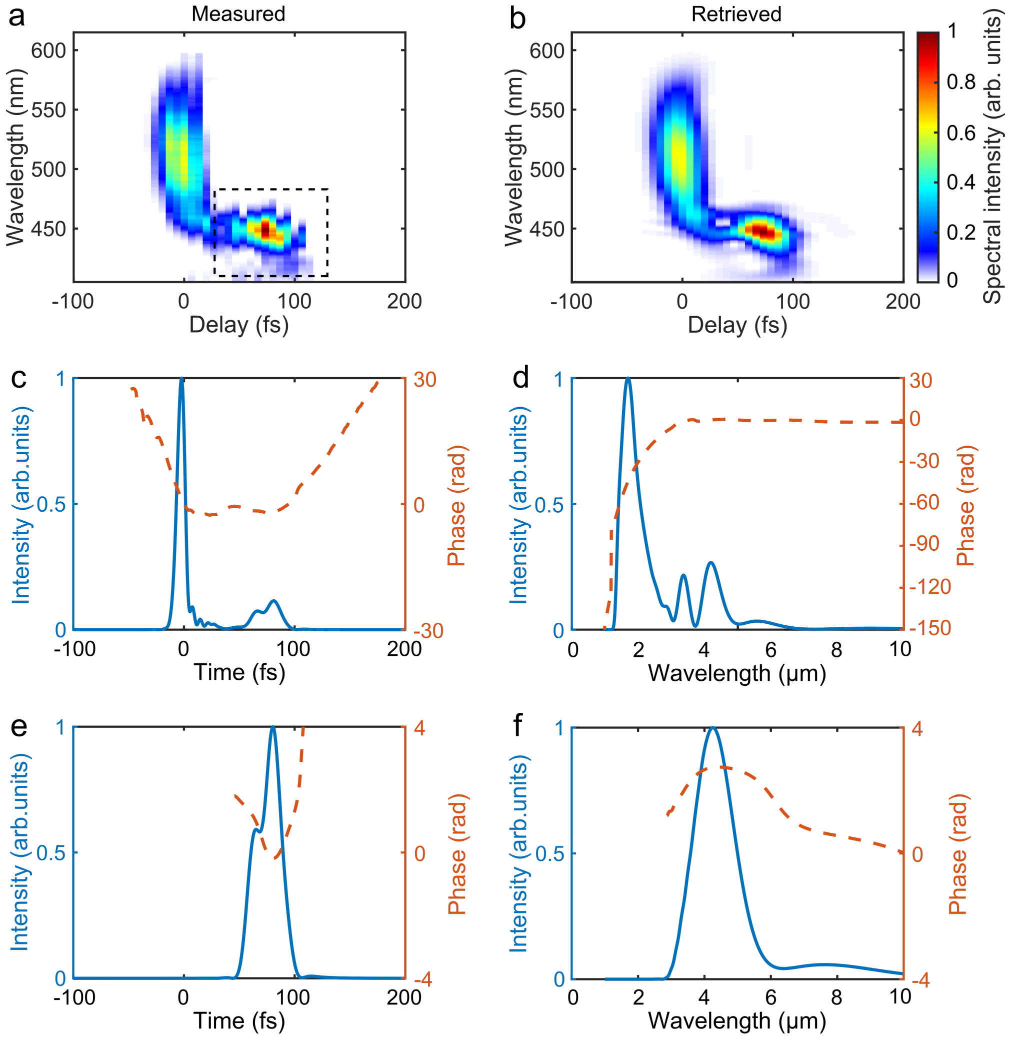

Supplementary Figure 12 The case with central wavelength of 4.2 μm. a,**b**, Measured and retrieved XFROG traces. **c**,**d**, Retrieved IR temporal and spectral intensity and phase. **e**,**f**, Retrieved temporal/spectral intensity and phase of the LWIR (dashed black box in (**a**)) pulse. The pulse duration (FWHM) of the LWIR pulse is 29.0 fs. The wavelength range (FWHM) of the LWIR pulse is 3.58-5.00 μm. The LWIR energy is 5.0 mJ. The estimated normalized vector potential $a_0 = 0.88$.

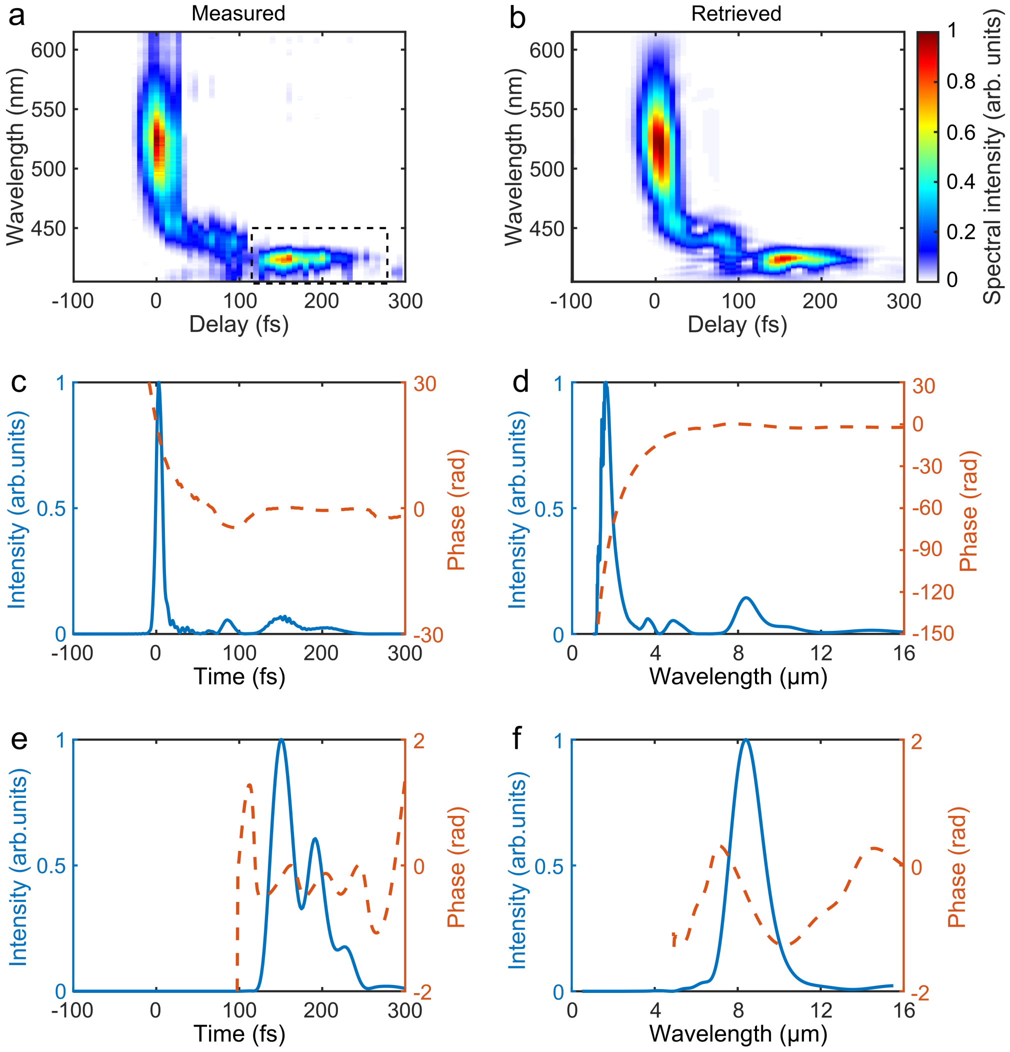

Supplementary Figure 13 The case with central wavelength of 8.3 μm. a,**b**, Measured and retrieved XFROG traces. **c**,**d**, Retrieved IR temporal/spectral intensity and phase. **e**,**f**, Retrieved temporal/spectral intensity and phase of the LWIR (dashed black box in (**a**)) pulse. The pulse duration (FWHM) of the LWIR pulse is 61.6 fs. The wavelength range (FWHM) of the LWIR pulse is 7.54-9.37 μm. The LWIR energy is 8.2 mJ. The estimated normalized vector potential a_0 = 1.54.

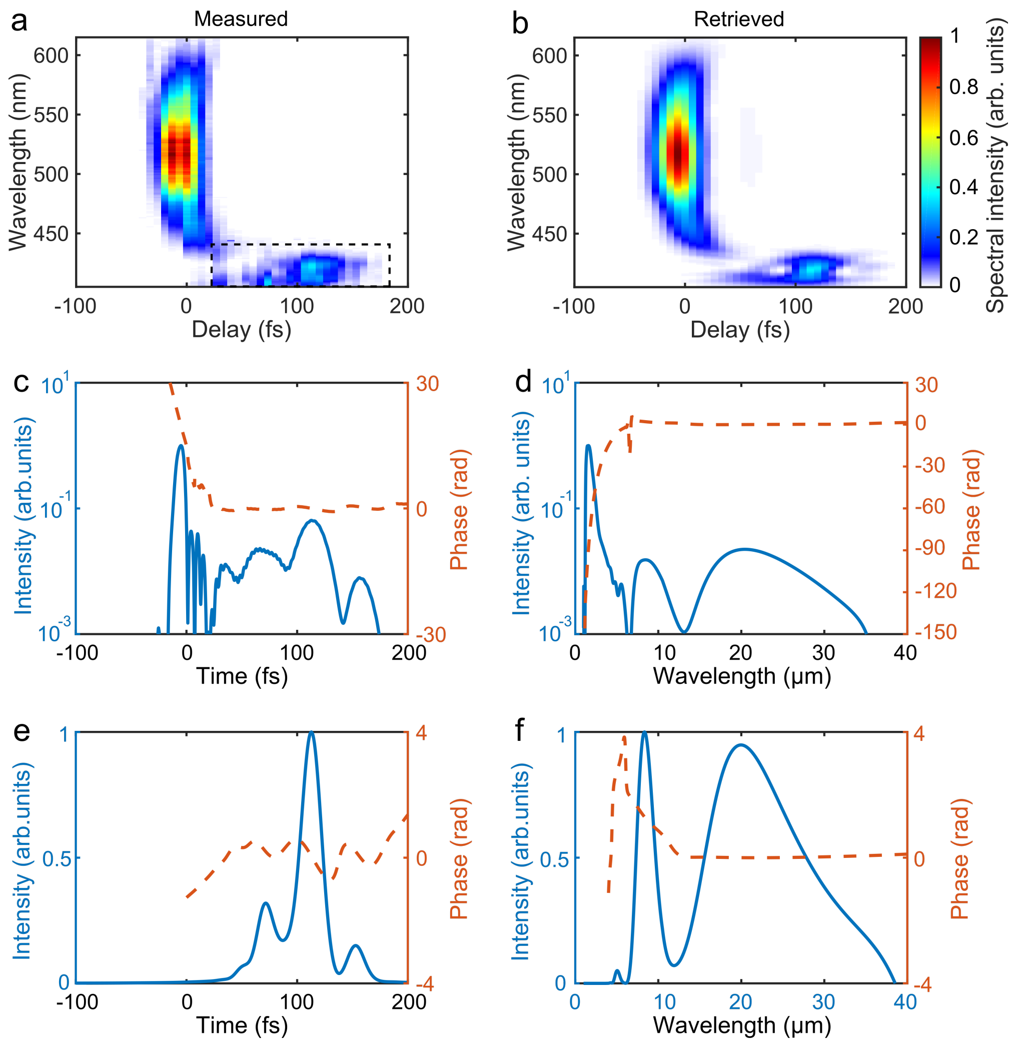

Supplementary Figure 14 The case with central wavelength of 20.0 μm. a,**b**, Measured and retrieved XFROG traces. **c**,**d**, Retrieved IR temporal/spectral intensity and phase. The intensities are shown using a logarithm scale. **e**,**f**, Retrieved temporal/spectral intensity and phase of the LWIR (dashed black box in (**a**)) pulse. In this case, the LWIR pulse has two wavelength peaks, with the 20.0 μm peak having more energy than the 8.4 μm peak. In practice, the LWIR pulse with the central wavelength of 20.0 μm can be extracted by a low-pass filter. Here, by numerical filtering, the pulse duration (FWHM) of this LWIR pulse is 77.4 fs. The wavelength range (FWHM) of the LWIR pulse is 15.4-28.3 μm. The LWIR energy is 5.7 mJ. The estimated normalized vector potential $a_0 = 2.75$.

Supplementary Note 1

Characterization of plasma density structures

In our experiments, the plasma density structures are produced using a round supersonic nozzle with an insertable blade that covers a portion of the gas jet. We combine online and offline measurements to fully characterize such non-axisymmetric plasma structures.

In the online measurement, the gas used is hydrogen. The delay between the ionization/wake generating pulse and the probe pulse of interferometry is around 50 ps. In such a short time period, the evolution of the shock profile (especially in the PC section) is negligible. The interferometry images of the plasma structure in three different cases (the blade inserts 275, 400, and 525 μm into the gas jet) are shown in Supplementary Fig. 1a-c. By Abel inversion, we can retrieve the plasma density profiles in the PC section, but not in the IR-CON and OC sections, since the plasma densities in these latter two sections are non-axisymmetric due to the oblique shock. The measured plasma density profiles in the PC sections are shown in Supplementary Fig. 1d. The mean plasma density in the plateau is about $7.2 \times 10^{18}\ \mathrm{cm}^{-3}$. To obtain the complete plasma structure information, we turn to the offline measurement.

In the offline measurement, hydrogen is replaced with argon to get a larger refractive index change. Using fluid simulations shown in Supplementary Fig. 2, one can see that the simulated density profiles of hydrogen and argon are similar with the same gas pressure, but the argon density is about 30% higher than the hydrogen density. A wavefront sensor (SID-4, PHASICS) camera is used to measure the phase difference

of the neutral argon gas at a particular gas jet setup at ten different angles. From these measurements, two-dimensional argon density profiles can be reconstructed by using a tomographic reconstruction algorithm (Supplementary Fig. 3). Therefore, the complete plasma density profile is obtained by multiplying the measured offline density profile (normalized to the plateau density in the PC section) with the measured online plateau density in the PC section ($7.2 \times 10^{18}\ \mathrm{cm}^{-3}$). (see Supplementary Fig. 4).

Supplementary Note 2

Correction of transport efficiencies and FWM efficiencies of IR pulses

In our experiment, the IR energy and XFROG signal (at a particular time) are measured simultaneously on every shot during the experiment. The measured IR energy (240 shots) for the case shown in Fig 2 is 133 ± 42 μJ as shown in Supplementary Fig. 6. To correctly retrieve the actually generated IR energy, two corrections need to be considered: transport efficiencies and FWM efficiencies for different IR wavelengths.

First, the net transport efficiency of the IR pulses from Gas jet 1 to Gas jet 2 can be estimated as follows. At the exit of Gas jet 1, all the IR beams with different wavelengths have roughly the same spot size of $w_0 = 13.5$ μm (the transverse wake size), and diffract like Gaussian pulses with divergent angles proportional to the wavelengths (confirmed by 3D-PIC simulations). Due to the limited collimation aperture (~35 mm) in the optical path, the collection efficiency decreases with the increase of the IR wavelength, and can be estimated using expressions for Gaussian beam propagation (for $\lambda < 2$ μm, the collection efficiency is close to 1). The total transport efficiency can be obtained by further including the transmission of every optical component in the transport path, which is either measured directly or supplied by manufacturers.

For the FWM efficiencies of XFROG at Gas jet 2, a calculation based on the integral equation method[1] is carried out for different IR wavelengths, where the effective length of Gas jet 2 is 1.5 mm, and the argon gas density is 8×10^{18} cm^{-3}. The focal spot size w is roughly proportional to the IR wavelength for $\lambda > 2$ μm ($w \propto$

$\frac{\lambda f}{D}$), where D is the beam size after collimation (~35 mm), and f is the focal length of the off-axis parabola. Based on the above parameters (and also the focal spot size of the reference beam 26.6 μm), the FWM phase-matching function $F^{(3)}$ (representing conversion efficiencies) of different IR wavelengths can be calculated, shown in Supplementary Fig. 7. Both the FWDFG and FWSFG efficiencies increase with the IR wavelength, with FWDFG more efficient than FWSFG for most wavelengths.

Combining the above two corrections, the corrected IR spectra at Gas jet 1 can be obtained. In Supplementary Fig. 8, a comparison of the normalized IR spectra before and after correction is shown to have relatively small differences, and this is mainly due to the complementary effect of the transport efficiency and the FWM efficiency.

In addition, the IR energy is simultaneously measured together with XFROG measurement. Similar transport efficiency correction is considered for this measurement. With the measurements of the IR energy and IR spectra, the full information of the generated IR pulse at Gas jet 1 can be properly retrieved.

Supplementary Note 3

Comparison of PIC simulation and experimental results

To make a direct comparison with the experimental result, full 3D PIC simulations are performed using the code OSIRIS[2,3]. The plasma density profile in the simulation is set to the measured density profile when the blade is inserted 400 μm into the gas jet. The drive laser in the simulation is an ideal Gaussian beam with the same spot size as the actual laser beam for simplicity. The laser energy in the simulation is set to 410 mJ so that it has the same enclosed energy as the actual drive laser beam.

The simulated transverse electric field of the original and filtered LWIR (6–20 μm) pulse after exiting the plasma structure (begin to diffract) are shown in Supplementary Fig. 9a and b, respectively. The simulated LWIR pulse energy in the wavelength range of 6–20 μm is 4.1 mJ, corresponding to a conversion efficiency of 1%. The simulated LWIR pulse has a central wavelength of 9.6 μm, a pulse duration (FWHM) of 36 fs (Supplementary Fig. 9c), and a near ideal TEM_{00} mode after exiting the plasma structure (Supplementary Fig. 9d). The simulation also gives us the electric field of the LWIR pulse as shown in Supplementary Fig. 9c. A direct comparison of the measured and simulated spectrum is shown to have reasonably good agreement (Supplementary Fig. 10).

For further improvement of the conversion efficiency, two main factors could be optimized. First, the driving laser's beam quality can be improved: In the experiment, only 60% of the laser energy is contained within the central spot size w_0=13.5±1.0 μm, corresponding to 70% of ideal Gaussian pump energy. Second, the plasma density

profile in the experiment is only an approximation of the ideal profile necessary for achieving the maximum conversion efficiency[4]. Specifically, the plasma downramp scale length is too long (~500 μm) in the present experiment. It needs to be shortened to ~100 μm to approach the theoretical efficiency.

Supplementary References

1. Hilber, G., Brink, D. J., Lago, A. & Wallenstein, R. Optical-frequency conversion in gases using Gaussian laser beams with different confocal parameters. *Phys. Rev. A* **38**, 6231–6239 (1988).
2. Fonseca, R. A. *et al.* OSIRIS: A Three-Dimensional, Fully Relativistic Particle in Cell Code for Modeling Plasma Based Accelerators. in *Computational Science — ICCS 2002* (ed. P. Sloot, A. Hoekstra, Dongarra, C. T. and J. D.) 342–351 (Springer Berlin / Heidelberg, 2002).
3. Fonseca, R. A. *et al.* One-to-one direct modeling of experiments and astrophysical scenarios: pushing the envelope on kinetic plasma simulations. *Plasma Phys. Control. Fusion* **50**, 124034 (2008).
4. Nie, Z. *et al.* Relativistic single-cycle tunable infrared pulses generated from a tailored plasma density structure. *Nat. Photonics* **12**, 489–494 (2018).
